# TGF-β1 accelerates the hepatitis B virus X-induced malignant transformation of hepatic progenitor cells by upregulating miR-199a-3p

**DOI:** 10.1038/s41388-019-1107-9

**Published:** 2019-11-18

**Authors:** Ke-shuai Dong, Yan Chen, Guang Yang, Zhi-bin Liao, Hong-wei Zhang, Hui-fang Liang, Xiao-ping Chen, Han-hua Dong

**Affiliations:** 1 Hepatic Surgery Center, Department of Hepatic Surgery, Tongji Hospital, Tongji Medical College, Huazhong University of Science and Technology, Wuhan, China; 2 Department of Hepatobiliary and Laparoscopic Surgery, Renmin Hospital, Wuhan University, Hubei Key Laboratory of Digestive System Disease, Wuhan, China; 3 Department of General Surgery, Tongji Hospital, Tongji Medical College, Huazhong University of Science and Technology, Wuhan, China

**Keywords:** Transdifferentiation, Cancer stem cells, Cancer stem cells, Transdifferentiation, Cancer stem cells

## Abstract

Increasing evidence has suggested that liver cancer arises partially from transformed hepatic progenitor cells (HPCs). However, the detailed mechanisms underlying HPC transformation are poorly understood. In this study, we provide evidence linking the coexistence of hepatitis B virus X protein (HBx) and transforming growth factor beta 1 (TGF-β1) with miR-199a-3p in the malignant transformation of HPCs. The examination of liver cancer specimens demonstrated that HBx and TGF-β1 expression was positively correlated with epithelial cell adhesion molecule (EpCAM) and cluster of differentiation 90 (CD90). Importantly, EpCAM and CD90 expression was much higher in the specimens expressing both high HBx and high TGF-β1 than in those with high HBx or high TGF-β1 and the double-low-expression group. HBx and TGF-β1 double-high expression was significantly associated with poor prognosis in primary liver cancer. We also found that HBx and TGF-β1 induced the transformation of HPCs into hepatic cancer stem cells and promoted epithelial–mesenchymal transformation, which was further enhanced by concomitant HBx and TGF-β1 exposure. Moreover, activation of the c-Jun N-terminal kinase (JNK)/c-Jun pathway was involved in the malignant transformation of HPCs. miR-199a-3p was identified as a significantly upregulated microRNA in HPCs upon HBx and TGF-β1 exposure, which were shown to promote miR-199a-3p expression via c-Jun-mediated activation. Finally, we found that miR-199a-3p was responsible for the malignant transformation of HPCs. In conclusion, our results provide evidence that TGF-β1 cooperates with HBx to promote the malignant transformation of HPCs through a JNK/c-Jun/miR-199a-3p-dependent pathway. This may open new avenues for therapeutic interventions targeting the malignant transformation of HPCs in treating liver cancer.

## Introduction

Hepatocellular carcinoma (HCC), the most common type of primary liver cancer, is a highly malignant disease and the third leading cause of cancer-related deaths worldwide [1]. Although great progress in its treatment has been achieved over recent decades, patient outcomes are still unsatisfactory [2]. The lack of effective therapeutic drugs can be attributed to a poor understanding of the complicated mechanisms of hepatocarcinogenesis. Extensive evidence has supported the notion that liver cancer partially arises from transformed hepatic progenitor cells (HPCs), which are putative liver stem cells with the bipotential capacity to differentiate into hepatocytes or cholangiocytes [3–6]. When the replicative capacity of hepatocytes is severely impaired through severe liver injury, activated HPCs emerge and expand from the canals of Hering and later invade the entire lobular parenchyma [7]. The HPC expansion is mainly caused by various pathologies, or environmental or genetic etiologies of liver cancer, such as fibrosis, cirrhosis, inflammation, and viral infection [4]. These factors also cause some HPC descendants to evolve into hepatic cancer stem cells (HCSC) with an unlimited self-renewal capacity and differentiation potential, resulting in the formation of a premalignant lesion [8]. However, the detailed mechanism by which these factors affect the properties of HPCs and control their evolution into HCSCs remains poorly unclear.

The development of HCC is closely related to the presence of chronic hepatitis B virus (HBV) infection. The multifunctional HBV X protein (HBx) encoded by HBV is essential for viral replication and HBV-induced carcinogenesis. HBx can alter the pattern of host gene expression, stimulate signal transduction, and inhibit the proteasomal degradation of growth regulatory proteins through interacting with signal transduction components or transcription factors [9, 10]. In addition, our group reported that HPCs have the capacity to cause HCC with the cooperation of the HBx gene and aflatoxin B1 (AFB1) in the liver microenvironment [11]. Moreover, HBx was shown to induce intrinsic cellular transformation which promoted the expansion and tumorigenicity of HPCs in 3,5-diethoxycarbonyl-1,4-dihydrocollidine (DDC)-treated mice [12]. HBx also triggered the malignant transformation of HepG2 cells by promoting characteristic HCSC properties [13]. Although its mechanism remains to be defined, HBx appears to be very important in HPC/HCSC-mediated liver tumors.

Transforming growth factor beta 1 (TGF-β1) is a multifunctional cytokine predominantly produced by activated mesenchymal cells in HBV-related liver injury [14]. It has been reported to be an important modulator of a broad spectrum of cellular processes, including polarity, growth, differentiation, wound repair, and apoptosis [15, 16]. TGF-β1 is also known to activate hepatic stellate cells and cause liver fibrosis, which contributes to hepatocarcinogenesis and tumor progression [17]. TGF-β1 plays a central role in epithelial–mesenchymal transition (EMT), which is a critical cellular event of tumor metastasis. Additional evidence suggests a potentially important role for TGF-β signaling in the regulation of HPCs and HCSCs [18, 19]. Hepatoma-initiating cells may derive from HPCs exposed to chronic and constant TGF-β1 stimulation [20], while HBx might induce the expression of TGF-β1 in the early stages of HBV infection [21]. HBx also shifts hepatocytic TGF-β signaling from the tumor-suppressive pSmad3C pathway to the oncogenic pSmad3L pathway in early carcinogenic processes [22]. Combined with the above effect of HBx on HPC-mediated tumorigenicity, these findings underscore the potentially close relationship between HBx, TGF-β1, and HPC transformation. However, the effect of these two risk factors on HPCs remains largely unknown.

MircroRNAs (miRNAs) are small non-coding RNAs composed of 20–25 nucleotides that silence cognate target genes through their complementary binding, cleavage of mRNAs, and translation inhibition [23]. miRNAs are involved in the regulation of developmental timing, embryogenesis, organogenesis, and the differentiation of stem cells and progenitor cells [24]. miR-122, the most abundant miRNA in the liver, is required for the progression of hepatocyte differentiation [25], while miR-194 in HPCs accelerated their differentiation into hepatocytes by targeting yes-associated protein-1 [26]. miR-139-3p and miR-199a-3p exert opposite effects on myeloid progenitor expansion and leukemic transformation [27]. Considering the potential common regulation of these miRNAs, we hypothesized that they might have crucial roles in the transformation of HPCs to promote tumorigenesis.

The present study, therefore, determined whether TGF-β1 synergistically functions with HBx to promote the malignant transformation of HPCs, and aimed to identify the miRNAs involved in this progression. We demonstrated that the simultaneous overexpression of HBx and TGF-β1 was significantly associated with HCSC properties and poor prognosis in primary liver cancer patients. TGF-β1 cooperated with HBx to promote the conversion of HPCs into HCSCs and induced EMT, leading to activation of the c-Jun N-terminal kinase (JNK)/c-Jun pathway. Furthermore, miR-199a-3p was identified as a regulator of HPC transformation, which could be transcriptionally activated by c-Jun. Our findings unveil a novel TGF-β1/HBx coregulated miR-199a-3p signaling axis in HPCs that may allow the development of novel therapeutic interventions for targeting the malignant transformation of HPCs.

## Results

### HBx and TGF-β1 expression positively correlates with HCSC marker expression and predicts a poor prognosis in HBV-related liver cancer

**Fig. 1:**
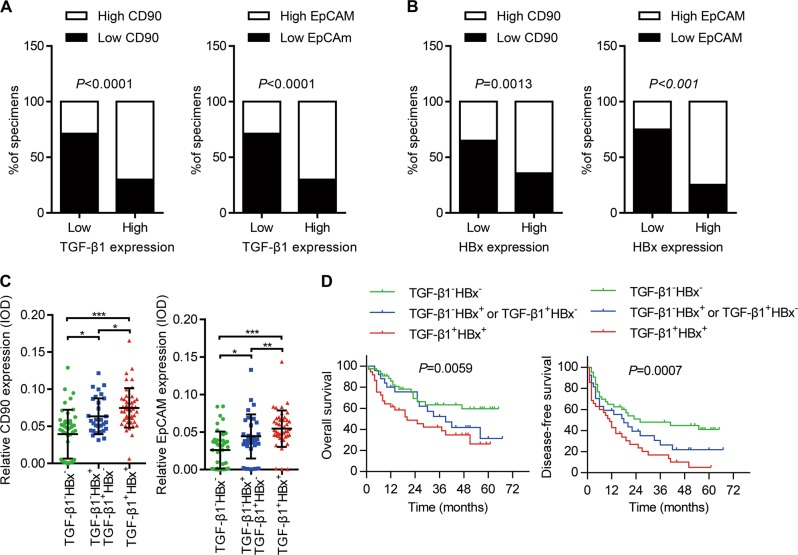
The levels HBx and TGF-β1 positively correlate with hepatic stem cell markers expression and predict poor prognosis in HBV-related liver cancer. **a, b** Consecutive patient HCC sections were applied for immunohistochemical (IHC) staining for expression of TGF-β1, HBx, CD90, and EpCAM (*n* = 119). The expression levels of HBx or TGF-β1 were both positively correlated with expression levels of CD90, EpCAM which are calculated by average integrated option density (IOD). *P* values were calculated by Pearson’s chi-square test. **c** A dot density plot illustrates the relative CD90 and EpCAM expression levels among indicated groups. Concomitant overexpression of HBx and TGF-β1 exhibited higher expression of CD90 and EpCAM. *P* values were calculated by Mann–Whitney U test, *P < 0.05, **P < 0.01, ***P < 0.001. **d** Kaplan–Meier curves were performed to compare the overall survival and disease-free survival between the indicated groups. *P* values represent log-rank testing of difference in cumulative survival

To investigate the functional crosstalk between TGF-β1 and HBx in HPC transformation and HCSC generation, we measured the expression of TGF-β1, HBx, and cancer stem cell (CSC) markers CD90 and EpCAM by IHC staining in a liver tumor tissue microarray. The tissue microarray contained 119 liver tumor specimens, including 112 HCCs, seven intrahepatic cholangiocarcinomas, and corresponding paratumor tissues. Patients were divided into high- and low-expression groups based on median expression levels of each marker determined by immunostaining integrated option density (IOD) scores. High TGF-β1 expression was significantly associated with liver cirrhosis (*P* = 0.040) and vascular invasion (*P* = 0.005). Patients with higher HBx or CD90 expression also had a higher probability of liver cirrhosis (*P* = 0.002 or *P* = 0.020, respectively). Moreover, high EpCAM expression was associated with positive AFP expression (*P* = 0.042), vascular invasion rate (*P* = 0.043), poor tumor differentiation (*P* = 0.020), and advanced tumor–node–metastasis stage (*P* = 0.002) (Table S1). We next determined whether TGF-β1 or HBx expression was associated with that of CSC markers in tumor samples. Liver tumors with higher TGF-β1 expression also tended to have higher CD90 expression (70.0%, 42/60, *P* < 0.001) and EpCAM expression (70.0%, 42/60, *P* < 0.001) (Fig. 1a and Fig. S1A). In addition, CD90 and EpCAM staining intensities were strong in specimens with high HBx expression (64.4%, 38/59, *P* = 0.02 and 74.6%, 44/59, *P* < 0.001). In contrast, only 35.0% (21/60) of samples with low-HBx expression showed high CD90 staining, and 25.0% (15/60) showed high EpCAM staining (Fig. 1b and Fig. S1A). This demonstrated significant coexpression correlations between TGF-β1 or HBx and HCSC markers including CD90 and EpCAM.

All 119 patients were then divided into three groups based on TGF-β1 and HBx expression: TGF-β1^+^HBx^+^ (*n* = 45), double-high-TGF-β1/HBx expression; TGF-β1^–^HBx^+^ or TGF-β1^+^HBx^–^ (*n* = 31), single-high TGF-β1/HBx expression; and TGF-β1^–^HBx^–^ (*n* = 43), double-low-TGF-β1/HBx expression. Compared with other groups, the TGF-β1/HBx double-high expression group exhibited higher CD90 and EpCAM expression (Fig. 1c). In addition, higher expression of CD90 and EpCAM was observed in hepatic tumors with vascular invasion compared with those without vascular invasion (Fig. S1B), supporting the idea that a synergistic effect of TGF-β1 and HBx is associated with HCSC marker expression and liver tumor metastasis. We next correlated our findings with patient survival. Patients in the TGF-β1/HBx double-high group had reduced overall survival time and disease-free survival time compared with those in TGF-β1/HBx single-high and TGF-β1/HBx double-low groups (*P* = 0.0059 and *P* = 0.0007, respectively) (Fig. 1d). Collectively, the analyses of tumor specimens suggested that a crucially functional synergy between TGF-β1 and HBx may be responsible for HCSC generation and poor clinical outcome in liver cancer patients.

### HBx and TGF-β1 synergistically induce the transformation of HPCs into HCSCs

**Fig. 2:**
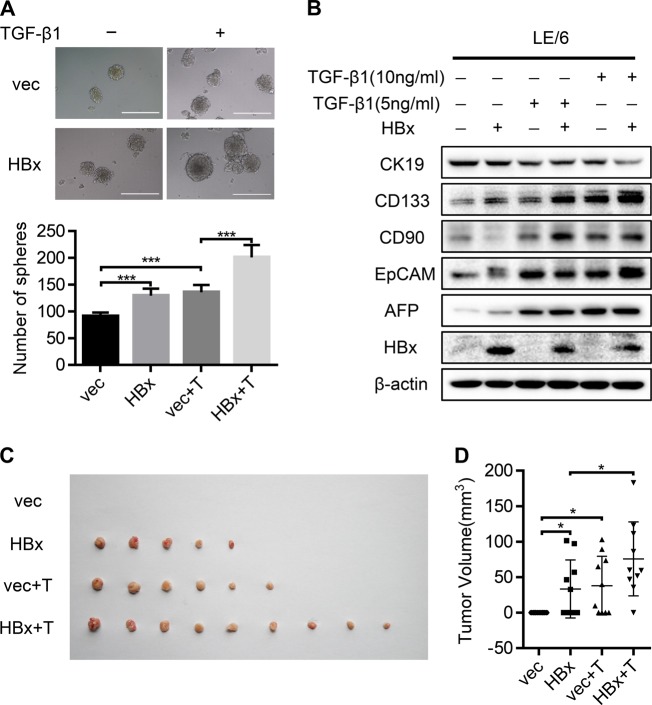
HBx and TGF-β1 induce the transformation of HPCs into HCSCs synergistically. **a** LE/6-vec, LE/6-HBx, LE/6-vec + T, and LE/6-HBx + T cells were seeded in non-attached plates to assay spheroid formation. Representative spheroids imaged under a phase-contract microscope(upper) and total numbers of spheroids from 1000 sorted cells (lower) are shown. Experiments were performed in triplicate and data are shown as mean ± SD. Scale bar, 200 μm. ***P < 0.001. **b** Western blots were performed to detect the expression of HBx, AFP, EpCAM, CD90, CD133, and CK19 in the above-indicated cells. **c** 1 × 10^6^ LE/6-vec, LE/6-HBx, LE/6-vec + T, and LE/6-HBx + T cells were subcutaneously injected into posterior flanks of the nude mice (*n* = 10). Xenografted tumors were excised 12 weeks postinoculation. Photograph of dissected tumors from each experimental group are presented. **d** Final tumor volumes are summarized in dot chart. Data represent mean ± SD. *P* values were calculated by Mann–Whitney U test. *P < 0.05

Considering that CSCs mostly derive from normal stem/progenitor cells in certain pathological microenvironments [20], we next explored the synergistic role of HBx and TGF-β1 in the transition of HPCs to HCSCs. The LE/6 cell line was transfected with either lentivirus containing HBx or lentivector. Western blot and qRT-PCR validated the overexpression of HBx after these lentiviruses were transfected into LE/6 cells (Fig. S2A, B). Subsequently, LE/6-vec and LE/6-HBx cells were treated with TGF-β1 for 8 weeks, then termed LE/6-vec + T and LE/6-HBx + T cells, respectively. We performed a spheroid formation assay to assess the self-renewal ability of HPCs. Compared with the vector control, LE/6-HBx cells formed spheroids more efficiently, which were further augmented by TGF-β1 exposure (Fig. 2a). Evaluation of the expression of five stemness-associated markers, including AFP, EpCAM, CD90, CD133, and CK19, in these four HPC cell lines revealed that HBx overexpression dramatically upregulated that of AFP, EpCAM, CD90, and CD133 (Fig. 2b). Furthermore, this effect was reinforced by TGF-β1 treatment. Immunofluorescence analysis also suggested that CD133 and EpCAM expression was much higher in LE/6-HBx + T cells than in other groups (Fig. S2C). Intriguingly, expression of CK19 and OV-6 HPC markers was significantly decreased after concomitant exposure to HBx and TGF-β1 (Fig. 2b and Fig. S2C). To explore the synergistic role of HBx and TGF-β1 in HPC tumorigenicity, nude mice were subcutaneously inoculated with LE/6-vec, LE/6-HBx, LE/6-vec + T, and LE/6-HBx + T cells. Tumor formation was observed in 0/10, 5/10, 6/10, and 9/10 mice, respectively, indicating that LE/6-HBx + T cells exhibited remarkably increased tumorigenicity (Fig. 2c, d). Consistent with our observations in cell lines, IHC staining showed increased AFP and CD90 expression, while CK19 expression was decreased in subcutaneous tumors arising from HBx-transfected cells with TGF-β1 treatment (Fig. S2E). Together, these data indicated that HBx and TGF-β1 contributed to the transformation of HPCs into HCSCs, which was further accelerated by concomitant exposure to HBx and TGF-β1.

### TGF-β1 cooperates with HBx to enhance the migration, invasion, and EMT of HPCs through the activation of c-Jun

**Fig. 3:**
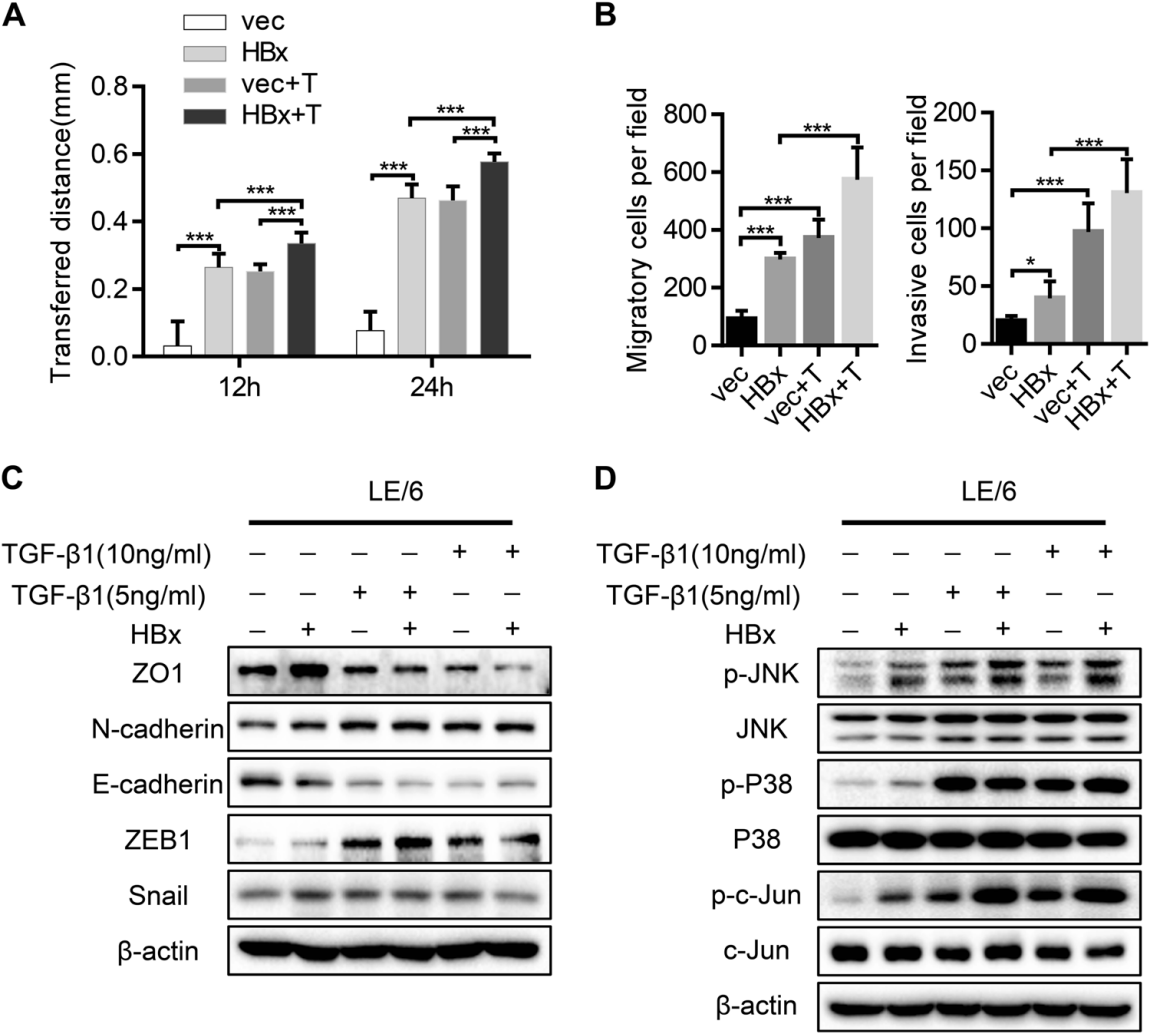
TGF-β1 cooperates with HBx to enhance the migration, invasion, and EMT of HPCs through the activation of c-Jun. **a** Wound healing assay was performed to measure the migration ability of the four cell lines as indicated. The histogram shows the transferred distance for the indicated cells. **b** Transwell assay was performed to assess the cell migration and invasion abilities in the four cell lines. Quantitative analysis of cell invasion and migration is shown. **c** LE/6-vec and LE/6-HBx cells were treated with TGF-β1 at 5 ng/ml or 10 ng/ml for 4 weeks. The expression of E-cadherin, N-cadherin, ZO1, ZEB1, and Snail was analyzed by western blot in the different cells. **d** Protein lysates from indicated cells were subjected to western blot analysis to detect the expression of indicated markers. Data represent mean ± SD. *P* values were calculated by Student’s *t* test. *P < 0.05, ***P < 0.001

HPCs were previously shown to maintain partial EMT state in vitro [28], and EMT induction provides CSCs with many specialized survival features to escape the primary site and disseminate throughout the body [29]. Therefore, we next clarified whether HBx and TGF-β1 were synergistically involved in regulating HPC migratory and invasive phenotypes. The cell wound healing assay was performed to assess cell motility, and HBx expression or TGF-β1 stimulation was shown to significantly increase the migratory speed of LE/6 cells, which was further accelerated by both HBx and TGF-β1 treatment (Fig. 3a and Fig. S3A). Migratory and invasive abilities were significantly increased when LE/6 cells were simultaneously exposed to HBx and TGF-β1 (Fig. 3b and Fig. S3B). Consistently, LE/6-HBx + T cells showed higher expression of ZEB1, and lower expression of E-cadherin and ZO1 compared with other cells (Fig. 3c). Intriguingly, Higher expression of N-cadherin was observed in LE/6 cells with HBx or TGF-β1 exposure. However, significant differences of N-cadherin expression were not demonstrated in LE/6-HBx + T cells when compared with those with TGF-β1 treatment only. The most plausible explanation was that compared with HBx, the TGF-β1 treatment had a stronger effect on the expression of N-cadherin. As a result, the effect of HBx was obscured. All these results indicated that TGF-β1 and HBx promoted migration, invasion, and EMT. HBx was reported to interact with the TGF-β signaling pathway [21, 22, 30], so we next examined the activation status of canonical and non-canonical downstream signaling of TGF-β. The non-canonical TGF-β pathway was activated in LE/6-HBx cells, as supported by the remarkable upregulation of p-JNK, p-P38, and p-c-Jun, but no significant change in p-Smad2 and p-Smad3 expression (Fig. 3d and Fig. S3C). The activation of the non-canonical TGF-β pathway was also observed in TGF-β1-treated cells. Notably, the phosphorylation level of JNK and c-Jun were further augmented by concomitant exposure to HBx and TGF-β1. Together, these data imply that the JNK/P38 pathway and subsequent c-Jun activation is at least partially involved in HPC transformation upon simultaneous exposure to HBx and TGF-β1.

### HBx and TGF-β1 exposure induces miR-199a-3p upregulation through the JNK/c-Jun pathway

**Fig. 4:**
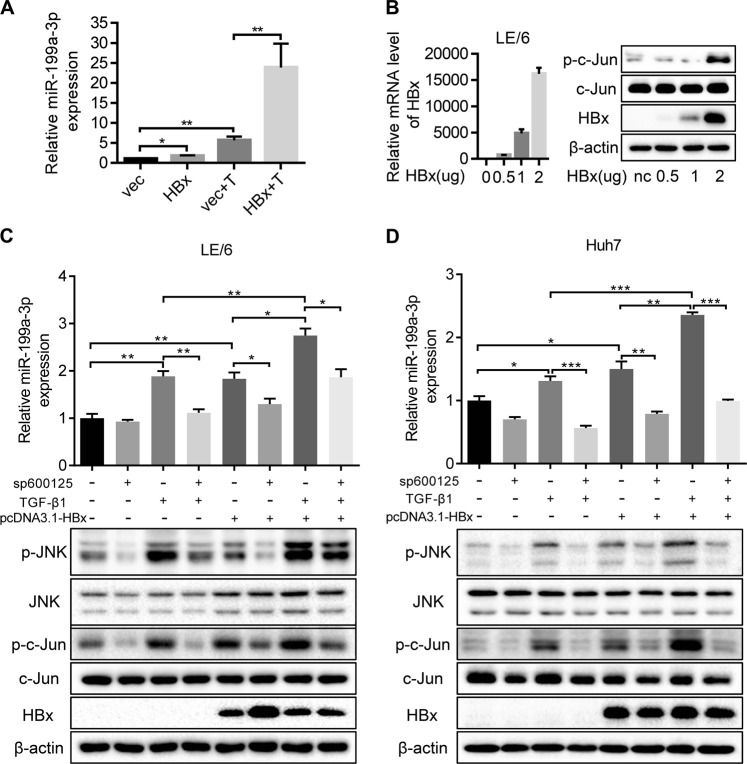
HBx and TGF-β1 exposure induce upregulation of miR-199a-3p through JNK/c-Jun pathway. **a** Comparison of miR-199a-3p expression in the indicated cells. **b** Left panel, HBx expression was confirmed by way of qRT-PCR in LE/6 cells treated with pcDNA3.1-HBx. Right panel, c-Jun and p-c-Jun expression were analyzed by western blot in LE/6 cells treated with pcDNA3.1-HBx. **c** LE/6 cells transiently transfected with pcDNA3.1-vec or pcDNA3.1-HBx were treated with/ without TGF-β1 or sp600125 for 24 h. The expression of miR-199a-3p were examined by qRT-PCR (upper) and the expression of HBx, c-Jun, p-c-Jun, JNK, p-JNK were determined by western blot (lower). **d** Huh7 cells transiently transfected with pcDNA3.1-vec or pcDNA3.1-HBx were treated with/ without TGF-β1 or sp600125 for 24 h. The expression of miR-199a-3p were examined by qRT-PCR (upper) and the expression of HBx, c-Jun, p-c-Jun, JNK, p-JNK were determined by western blot (lower). *n* = 3 per group, data represent mean ± SEM, *P* values were calculated by Student’s *t* test. *P < 0.05, **P < 0.01, ***P < 0.001

Because miRNAs play key roles in the regulation of CSC self-renewal and promote differentiation to determine CSC fates [31], we determined which miRNAs were differentially expressed in HBx-stably overexpressing LE/6 cells upon TGF-β1 exposure. We previously used an Agilent miRNA Base 16.0 microarray to identify 45 significantly differentially expressed miRNAs between HBV-related HCC patients and healthy volunteers [32]. Considering that an abundance of miRNA is crucial for its function, we quantified the top 14 most abundantly expressed miRNAs from the microarray by qRT-PCR in LE/6-stable transfectants with or without TGF-β1 exposure (Fig. S4A). In LE/6-HBx + T cells, the expression of miR-215-5p, miR-374a-5p, miR-188-5p, and miR-199a-3p was significantly elevated relative to that of other cells. miR-199a-3p was maximally affected by HBx and TGF-β1 exposure (Fig. 4a). We also found that TGF-β1 and HBx together increased the stemness and invasion ability of HPCs through the activation of c-Jun. In addition, transfection of pcDNA3.1-HBx into LE/6 cells triggered the activation of c-Jun (Fig. 4b), which was consistent with previous findings [33]. To further elucidate the relationship between c-Jun and these miRNAs, sp600125, an inhibitor of JNK that blocks JNK-mediated activation of c-Jun, was added to LE/6 cells transiently transfected with HBx and treated with TGF-β1. As shown in Fig. 4c and Fig. S4B, only miR-199a-3p was significantly elevated in response to HBx or TGF-β1 stimulation, and this was partially prevented by treatment with sp600125 which significantly inhibited the phosphorylation of JNK and subsequent phosphorylation of c-Jun. This effect was validated in Huh7 cells (Fig. 4d). Furthermore, knockdown of c-Jun by siRNA attenuated the upregulation of miR-199a-3p upon HBx or TGF-β1 stimulation in LE/6 cells when compared with siRNA negative controls (Fig. S4D). Interestingly, HBx/TGF-β1 could still lead to the increased expression of miR-199a-3p in LE/6 cells with c-Jun knockdown (Fig. S4D). A potential reason for this effect is that the c-Jun activation rather than total c-Jun is responsible for the expression of miR-199a-3p. Altogether, these results support the notion that HBx and TGF-β1 drive the upregulation of miR-199a-3p through a c-Jun-dependent mechanism.

### HBx and TGF-β1 activate the miR-199a-3p promoter through the transcriptional factor c-Jun

**Fig. 5:**
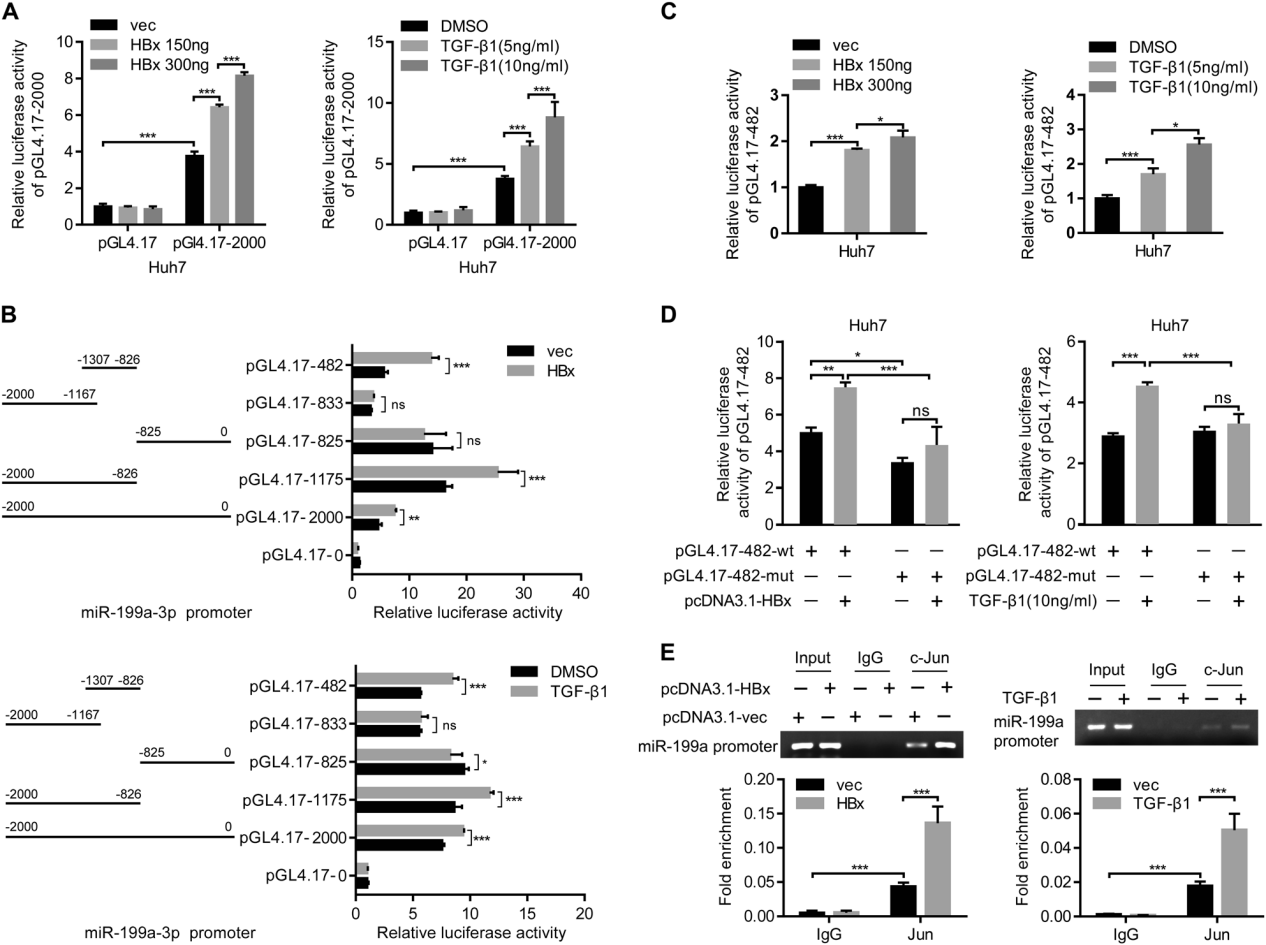
HBx and TGF-β1 activate miR-199a-3p promoter through transcriptional factor c-Jun. **a** Relative luciferase activities of pGL4.17 and pGL4.17-2000 were measured in Huh7 cells transfected with pcDNA3.1 vector or pcDNA3.1-HBx (left) and treated with or without TGF-β1 (right). **b** Schematic of miR-199a-3p promoter constructs. Relative luciferase activities of pGL4.17 containing various length of miR-199a-3p promoter regions were determined in Huh7 cells exposed to HBx (upper) and TGF-β1(lower). **c** Huh7 cells were cotransfected with pGL4.17-482 and pcDNA3.1-HBx at different doses, and luciferase activities were analyzed. Huh7 cells were transfected with pGL4.17-482 and then treated with TGF-β1 at different doses, and luciferase activities were analyzed. **d** Luciferase activities of wild-type (pGL4.17-482) or c-Jun response element mutant (pGL4.17-482-mut) miR-199a-3p promoter were examined in Huh7 cells cotransfected with control vector or pcDNA3.1-HBx (left) and treated with or without TGF-β1 (right). **e** Huh7 cells were transiently transfected with control vector or pcDNA3.1-HBx (left) and treated with or without TGF-β1 (right). The cell lysates were subjected to chromatin immunoprecipitation (ChIP) analysis with immunoglobin G or anti-c-Jun antibody. Pull-down DNA was analyzed by qRT-PCR using specific primer targeting the c-Jun binding site in miR-199a-3p promoter. *n* = 3 per group, data represent mean ± SD (**a–d**) or mean ± SEM (**e**), *P* values were calculated by Student’s *t* test. *P < 0.05, **P < 0.01, ***P < 0.001

HBx was previously shown to interact with DNA-binding proteins as a coactivator of transcription and to indirectly stimulate transcription by activating cellular signal transduction pathways. TGF-β activates Smad-independent signaling cascades, including Erk, JNK, and p38 MAPK pathways, then phosphorylates multiple transcription factors such as Stat3, ATF-2, and c-Jun, which are involved in regulating the expression of target genes [34–37]. Therefore, we next examined the effect of HBx and TGF-β1 on the activity of the miR-199a-3p promoter. The promoter region, covering 2.0 kb upstream of the miR-199a-3p transcriptional initiation site, was cloned. Luciferase reporter assays showed that the ectopic expression of HBx activated the full-length miR-199a-3p promoter (pGL4.17-2000) in a dose-dependent manner (Fig. 5a). Similarly, the luciferase activities of the full-length promoter were dose-dependently increased by TGF-β1 treatment (Fig. 5a). These results implied that HBx and TGF-β1 upregulated miR-199a-3p by controlling its transcription. The above results have demonstrated that TGF-β1 and HBx synergistically promote the activation of c-Jun and induce the upregulation of miR-199a-3p. Given that c-Jun functions as a transcriptional activator that can be enhanced by HBx [33], we speculated that c-Jun interacted with the miR-199a-3p promoter to activate its transcription, and that HBx or TGF-β1 promoted miR-199a-3p transcription by activating c-Jun. Strikingly, miR-199a-3p promoter analysis via PROMO and JASPAR revealed five highly conserved c-Jun response elements (data not shown). To map the c-Juninteracting region in the miR-199a-3p promoter, we generated several luciferase reporter plasmids of various miR-199a-3p promoter truncated mutants. Each was cotransfected together with pcDNA3.1-HBx into Huh7 cells, and the region from –1307 to –826 (pGL4.17-482) was shown to be critical for HBx-mediated transcriptional activation of miR-199a-3p (Fig. 5b). Similar results were observed in cells that were transfected with various truncated mutants and then treated with TGF-β1 (Fig. 5b). Moreover, the luciferase activities of pGL4.17-482 were remarkably increased by either HBx or TGF-β1 exposure in a dose-dependent manner (Fig. 5c).

To further validate this, we constructed a mutant of the promoter 482 region at the c-Jun response element (Fig. S5). Either HBx or TGF-β1 was found to enhance the miR-199a-3p promoter-luciferase activity, but not that of the promoter containing a c-Jun response element mutation (Fig. 5d). In addition, ChIP assays revealed that c-Jun was recruited to the miR-199a-3p promoter region, and HBx and TGF-β1 further enhanced the interaction of c-Jun with the miR-199a-3p promoter (Fig. 5e). Together, these results yield strong evidence that HBx and TGF-β1 lead to JNK-dependent c-Jun activation, then activated c-Jun is recruited to the miR-199a-3p promoter to promote miR-199a-3p transcription.

### miR-199a-3p contributes to the malignant transformation of HPCs

**Fig. 6:**
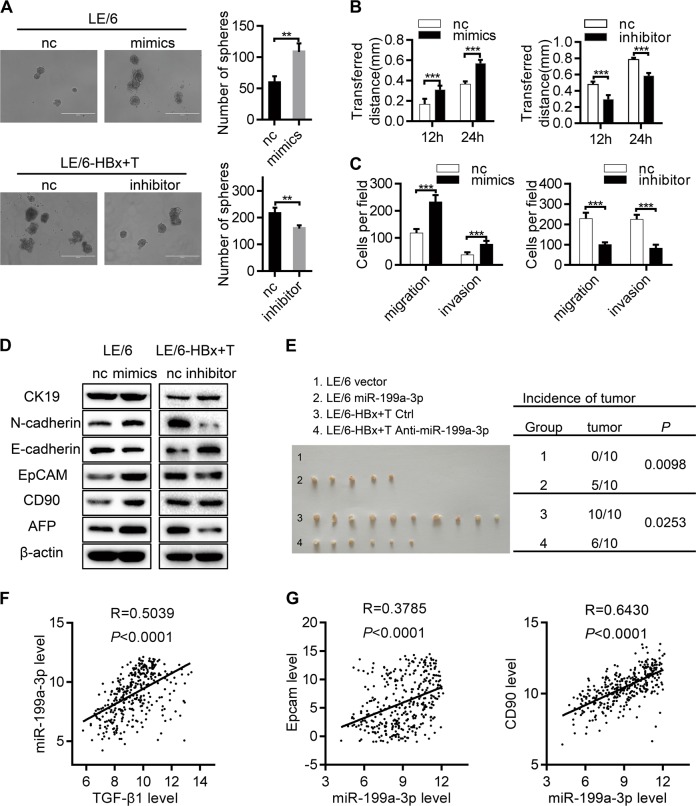
miR-199a-3p contributes to the malignant transformation of liver progenitor cells. **a** Spheroid formation assay of cells indicated was performed and the numbers of spheroids were counted. Scale bar, 400 μm. **b** Migration ability of LE/6 cells transfected with miR-199a-3p mimics or negative control and LE/6-HBx + T cells transfected with miR-199a-3p inhibitor or negative control was assessed by wound healing assay and quantified by measuring transferred distance at time point 12 h and 24 h. **c** Transwell assay was carried out to evaluate the cell migration and invasion abilities in the indicated cells. **d** The expression levels of AFP, CD90, EpCAM, E-cadherin, N-cadherin, and CK19 in the above-indicated cells were determined by western blot. **e** LE/6 stably overexpressing miR-199a-3p, LE/6-HBx + T with miR-199a-3p knockdown and the control vectors were subcutaneously injected into posterior flanks of the nude mice (*n* = 10) for 6 weeks. Subcutaneous tumors derived from indicated cells are presented and statistical analysis of the incidence of tumors is shown. **f** Coexpression correlation analysis was used to determine the correlations between TGF-β1 and miR-199a-3p in 357 human HCC specimens. Data was obtained from the TCGA database. **g** Coexpression correlation analysis was used to further determine the correlations between miR-199a-3p and CD90, EpCAM in 357 human HCC specimens from the same database. Data represent mean ± SD, *P* values were calculated by Student’s *t* test (**a–c**), Pearson’s χ2 (**e**) or Pearson’s correlation coefficient (**f, g**). *P < 0.05, **P < 0.01, ***P < 0.001

To gain insights into the pathophysiological role of miR-199a-3p in the transformation of HPCs, LE/6 cells with low miR-199a-3p expression were used in gain-of-function studies with miR-199a-3p mimics, and LE6/-HBx + T cells with high miR-199a-3p expression were used in loss-of-function studies with miR-199a-3p inhibitors. Ectopic miR-199a-3p overexpression significantly enhanced the self-renewal ability of LE/6 cells in the spheroid formation assay, while the opposite was observed in LE/6-HBx + T cells with miR-199a-3p knockdown (Fig. 6a). The wound-healing assay showed that miR-199a-3p overexpression augmented the migratory speed, and miR-199-3p knockdown reduced it (Fig. 6b and Fig. S6A). In addition, exogenous miR-199a-3p expression significantly enhanced migration and invasion capacities, while miR-199a-3p inhibition induced potent migration and invasion suppression (Fig. 6c and Fig. S6B). Western blot analysis further showed that miR-199a-3p strongly potentiated the expression of AFP, CD90, EpCAM, and N-cadherin and suppressed that of E-cadherin, whereas miR-199a-3p inhibition resulted in the inverse expression pattern (Fig. 6d), indicating that miR-199a-3p induced a stem cell-like property and EMT state in HPCs. In addition, LE/6 stably overexpressing miR-199a-3p, LE/6-HBx + T with miR-199a-3p knockdown and the control vectors were generated (Fig. S6C) and subcutaneously inoculated into the flanks of nude mice. The tumorigenicity assay showed that tumor formation was observed in 5/10 mice after inoculation with LE/6 cells stably overexpressing miR-199a-3p, whereas, no tumor was formed in those with LE/6 vector inoculation. Consistently, knockdown of miR-199a-3p significantly attenuated the tumorigenicity of LE/6-HBx + T cells (Fig. 6e). Finally, to explore the possible clinical relevance of miR-199a-3p, we investigated its expression and that of TGF-β1 in human liver tumor tissues from The Cancer Genome Atlas (TCGA) database. As shown in Fig. 6f, TGF-β1 was positively associated with miR-199a-3p expression. Further analysis revealed significant coexpression correlations between miR-199a-3p and CD90 and miR-199a-3p and EpCAM in the same clinical cohort of the TCGA database, providing strong clinical evidence for a critical role of miR-199a-3p in HCSC generation (Fig. 6g). Overall, these results suggest that miR-199a-3p functionally promotes the malignant transformation of HPCs.

## Discussion

**Fig. 7:**
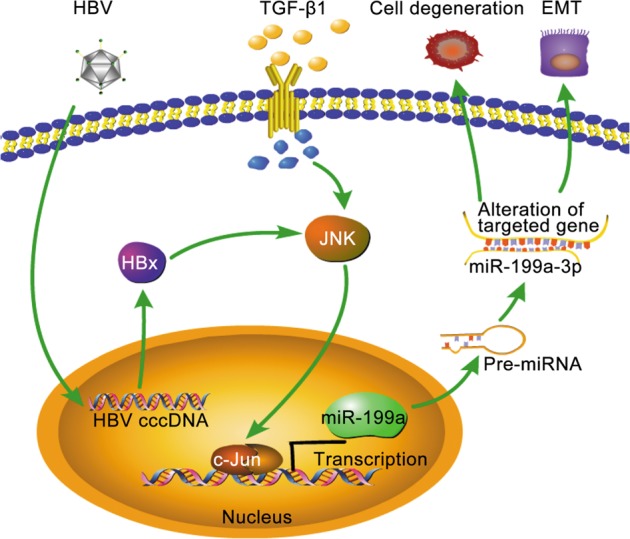
Schematic diagram of the proposed model in the HPCs. TGF-β1 acts to reinforce the degeneration and EMT induced by HBx through activating c-Jun then results in a transcriptional upregulation of the miR-199a-3p which could involve in the malignant transformation of HPCs

Current evidence suggests that primary liver cancers derive from mature hepatocytes subjected to reprogramming events that result in CSC features, or from the transformation of progenitor cells [38]. Such cells with genetic or epigenetic modifications are susceptible to malignant transformation following dysregulation of the microenvironments underlying the maintenance of normal homeostasis [38]. We previously showed that HPCs have the capacity to generate HCC with the cooperation of HBx and AFB1 in the liver microenvironment [11]. Here, we provided the first evidence linking the coexistence of HBx and TGF-β1 to the malignant transformation of HPCs. Our data revealed that TGF-β1 collaborated with HBx to induce the transformation of HPCs into HCSCs and promote EMT. Moreover, the activation of c-Jun was involved in the malignant transformation of HPCs. We also identified miR-199a-3p as a key regulator of HPC transformation upon HBx and TGF-β1 exposure, which induced the transcription of miR-199a-3p by activating c-Jun. Thereby, we reveal a novel TGF-β1/HBx-JNK/c-Jun-miR-199a-3p signaling axis that is pivotal to the malignant transformation of HPCs (Fig. 7).

The aberrant expression of HBx and TGF-β1 is frequently detected in liver tumors and plays an important role in the pathogenesis of fibrosis in chronic hepatitis and cirrhosis and in the development of HCC. Previous studies have reported functional crosstalk between HBx and TGF-β1 in HBV pathogenesis. For example, HBx transactivated the TGF-β1 promoter [21], interacted with Smad4 [39], and downregulated PPM1a [40], thereby amplifying TGF-β1 signaling. HBx shifted hepatocytic TGF-β1 signaling from tumor suppression to oncogenesis in the early carcinogenic process [22], and the TGF-β1 pathway may be involved in the accelerated tumor development of HBx transgenic mice after partial hepatectomy [30]. These studies indicate that HBx participates in HBV-related pathogenesis and aggravates carcinogenesis in cooperation with TGF-β1. We previously found that HBx promoted abnormal oval cell differentiation and induced malignant transformation [11]. Subsequently, HBx was shown to increase the expansion and tumorigenicity of HPCs, which contributed to HBx-mediated tumor formation in a DDC-induced mouse model [12]. Moreover, TGF-β1 exposure induced the transformation of HPCs and gave rise to the initiation of tumor cell characteristics [20]. Although these reports showed that HBx or TGF-β1 may contribute to HPC-mediated liver tumors, HPC changes that occur in the presence of HBx and TGF-β1 have not been investigated. This study extended previous observations and revealed a strongly synergistic function of HBx and TGF-β1 in the malignant transformation of HPCs.

HCSCs are a subset of cells with stem cell features that are responsible for tumor initiation, relapse, metastasis, and chemoresistance [8]. Experimentally, putative HCSCs have been isolated using CD90, EpCAM, CD133, AFP, OV-6, and CK19 cell surface markers that are also expressed by normal progenitor cells [41–44]. Analysis of clinical specimens in the present study demonstrated that HBx and TGF-β1 expression was positively correlated with that of EpCAM and CD90. More importantly, EpCAM and CD90 expression was much higher in the HBx and TGF-β1 double-high expression group than the single-high or double-low groups. Moreover, HBx and TGF-β1 double-high expression was significantly associated with poor prognosis in primary liver cancer patients, indicating that the coexistence of HBx and TGF-β1 may be responsible for the generation of HCSCs.

The origin of HSCSs is thought to derive from the transformation of normal HPCs [38]. Stem cell-like features of HCSCs from transformed HPCs were confirmed by functional in vitro clonogenicity and in vivo tumorigenicity assays. Here, our data indicated that HBx increased the expression of CD90, EpCAM, CD133, and AFP and enhanced the self-renewal ability of HPCs, and that these effects could be further accelerated by TGF-β1. Unlike HBx and TGF-β1-stimulated HPCs, which expressed high levels of CSC markers AFP, CD90, and EpCAM, normal HPCs did not form tumors upon subcutaneous inoculation into nude mice. Notably, specific HPC surface markers OV-6 and CK19 exhibited the opposite trend, which is consistent with a previous study [20]. These results suggest that the expression patterns of various stem cell markers in HCSCs may be different, possible because of the heterogeneity of activated HPC signaling pathways.

EMT is a fundamental process of development and disease progression. It describes a series of events during which epithelial cells lose many of their characteristics and take on the properties of mesenchymal cells, requiring complex changes in cell architecture and behavior [45]. Some epithelial cells use components of the EMT program as the main route for acquiring self-renewing traits of stem cells [46]. Mani et al. provided evidence that untransformed human mammary epithelial cells acquired stem cell-like characteristics through an EMT process that promoted the generation of cancer stem cells from more differentiated neoplastic cells [47]. Recently, Huang et al. reported that Tg737 influenced the malignant transformation of HPCs to HCSCs by regulating the β-catenin/Snail/HNF4α feedback circuit, which promoted EMT. However, whether EMT is also involved in the transformation of HPCs into HCSCs induced by HBx was largely unknown. Here, we found that TGF-β1 together with HBx conferred mesenchymal attributes to HPCs, including enhanced cell migration and invasion abilities and the expression of EMT-representative markers. HPCs appear to enter into an HCSC state, depending on the EMT involvement. c-Jun has been implicated in the EMT process and the maintenance of cancer stemness [48, 49]. In this study, the JNK/P38 pathway and subsequent c-Jun activation were observed in HPCs upon HBx and TGF-β1 exposure. Because the enrichment of c-Jun and Fos activity was detected in the hepatoblast subtype of HCC arising from bipotential HPCs, this indicated that the AP-1 complex was the major driving force in tumorigenesis of the hepatoblast subtype [3]. Moreover, positive feedback regulation of OCT4 and c-Jun, resulting in the continuous expression of c-Jun, was critical for the induction of CSC-like characteristics in liver cancer [49], as seen in other cancers [50, 51]. These results illustrate that the activation of c-Jun in HPCs is responsible for their malignant transformation.

Increasing evidence demonstrates that miRNAs participate in the regulation of self-renewal, differentiation, and transformation in normal stem cells and CSCs [52]. Specifically, the downregulation of miR-200a induced EMT phenotypes and CSC-like signatures through targeting the β-catenin pathway in HPCs [52], while miR-216b-mediated PTEN suppression was involved in and HCSC generation in HPCs exposed to TGF-β1 [20]. We found that HBx and TGF-β1 together induced significant upregulation of miR-199a-3p, which was partially blocked by c-Jun inhibition, implying that miR-199a-3p might be responsible for HBx and TGF-β1-induced HPC transformation. However, the underlying mechanisms by which TGF-β1 cooperate with HBx to regulate miR-199a-3p expression require further exploration. HBx was shown to function as a transcriptional coregulator that modulated the expression of multiple genes through binding to transcription factors [53]. Our group demonstrated that HBx promoted cAMP response element-binding protein-mediated activation of miR-3188 and Notch signaling in HCC [54]. Notably, HBx and Jab1 interacted in the cytoplasm to enhance the phosphorylation of JNK and c-Jun, with subsequent activator protein-1 activation [33]. On the other hand, JNK-mediate phosphorylation activated by TGF-β increase the transcriptional efficiency of c-Jun by strengthening its binging to gene promoters [36, 37]. Accordingly, our data showed that HBx or TGF-β1 promoted miR-199a-3p expression via c-Jun-mediated activation.

We also provided evidence for an important role of miR-199a-3p in promoting the malignant transformation of HPCs into HCSCs, which was in agreement with the findings of Alemdehy et al. They identified an oncogenic function of miR-199a-3p in acute myeloid leukemia through enhancing proliferation and self-renewal of myeloid progenitors [27]. Celià-Terrassa et al. also reported that miR-199a-3p was upregulated in breast CSC populations and induced stem cell-like signatures [55]. Notably, miR-199a-3p was reported to have either tumor-suppressive functions [56, 57] or tumor-promoting activities [27, 58] across different cancer types, including liver cancer. These apparent opposing roles of miR-199a-3p in cancer development is perhaps not surprising given its multiple downstream targets and the complexity of microenvironmental factors that regulate cell properties during different stages of tumor progression. It would, therefore, be worthwhile investigating whether miR-199a-3p consistently regulates progenitor cells in other cancers. Furthermore, our current data do not elucidate a downstream target gene of miR-199a-3p, so future studies should focus on the detailed mechanism underlying the role of miR-199a-3p in HPC transformation.

In summary, our results provided novel evidence that TGF-β1 cooperates with HBx to promote the malignant transformation of HPCs through a JNK/c-Jun/miR-199a-3p-dependent pathway. Understanding the key functional pathways that regulate HPC transformation could lead to strategies to suppress hepatocarcinogenesis.

## Materials and methods

### Clinical specimens and data

Human primary liver cancer tissue samples were obtained from patients who underwent liver resection at the Hepatic Surgery Center of Tongji Hospital and who provided their informed consent. Tissue microarrays from a total of 119 formalin-fixed paraffin-embedded samples were used for immunohistochemical (IHC) analyses. Patient clinical characteristics are shown in Table S2. This study was approved by the Medical Ethics Committee of Tongji Hospital and was conducted according to the Declaration of Helsinki. Written informed consent for data analysis was obtained from all patients before operation.

### Cell culture and transfection

Hepatic stem-like epithelial cells (LE/6) were a generous gift from Prof. Nelson Fausto. They were cultured in Dulbecco’s minimal essential medium: Ham’s F-10 (1:1) (Invitrogen, Waltham, MA) supplemented with 1 μg/ml insulin (Sigma, St Louis, MO) and 0.5 μg/ml hydrocortisone (Sigma). 293 T and Huh7 cell lines were purchased from the China Center for Type Culture Collection (Wuhan, China), and maintained in DMEM (Gibco, USA) medium. All cell cultures were supplemented with 10% fetal bovine serum (Gibco), 100 IU/ml penicillin and streptomycin in 5% CO_2_ at 37 °C. Lentivirus was packaged in 293 T cells. 48 h after the cotransfection, virus-containing supernatants were collected and incubated with LE/6 cells in the presence of 10 μg/ml polybrene for 24 h. HBx-stably overexpressing (LE/6-HBx) and control (LE/6-vec) cells were achieved by selection with 5 μg/ml puromycin for 2 weeks. LE/6-vec and LE/6-HBx cells were then treated with 5 ng/ml or 10 ng/ml TGF-β1 for 8 weeks, and renamed LE/6-vec + T and LE/6-HBx + T cells, respectively. Cells stably with miR-199a-3p overexpression or knockdown and the control vectors were generated by selection with 500 μg/ml hygromycin or neomycin for 2 weeks.

### IHC staining

IHC analysis of clinical tumor samples and tumor xenograft samples was performed using antibodies against HBx, TGF-β1, CD90, epithelial cell adhesion molecule (EpCAM), α-fetoprotein (AFP), and CK19. Image acquisition was performed using the 3DHIESTECH scan system and software. The cell-based average IOD for individual protein expression in serial tumor sections for each sample was analyzed by Image-Pro Plus 6.0 software (Media Cybernetics Inc., Bethesda, MD). The cutoff for the definition of high or low expression was the median value. Accordingly, samples were segregated into two groups for each analysis. Each data set was analyzed separately and a consensus evaluation from at least two of the three investigators was considered acceptable.

### Western blot

Cells were washed once with cold PBS and lysed in RIPA buffer supplemented with 1% protease and 1% phosphatase inhibitor cocktail. The protein concentrations were determined by BCA assay. Subsequently, proteins in the same amount were separated by sodium dodecyl sulfate-polyacrylamide gel electrophoresis (SDS-PAGE) and transferred onto polyvinylidene fluoride (PVDF) membranes. Then, the membranes were blocked in 5% skim milk for 2 h and then incubated with appropriate primary antibody at 4 °C overnight. Next, horseradish peroxidase-linked secondary antibody such as goat anti-rabbit or anti-mouse antibody were incubated with the blots. Bio-Rad GelDoc system was used for signal detection. The antibodies used are listed in Table S3.

### Real-time PCR

Total RNA was extracted using Trizol regent (Invitrogen), and reverse transcription was performed from 2 μg total RNAs using a FastQuant cDNA Synthesis Kit or miRcute Plus miRNA First-Strand cDNA Synthesis Kit (TIANGEN, Beijing, China). Quantitative real-time PCR was carried out on a CFX Connect™ Real-Time PCR Detection System (Bio-Rad, Hercules, CA, USA) with SuperReal PreMix Plus or miRcute miRNA qPCR Detection Kit (SYBR Green, TIANGEN, Beijing, China) according to the manufacture’s instruction. The quantity of mRNA and miRNA was calculated using ΔΔCt method and GAPDH and U6 were used as controls. All reactions were performed as triplicates. The primer sequences are listed in Table S4.

### Xenograft tumor formation

Male BALB/c nude mice (4–6 weeks old) were purchased from Huafukang Bioscience Co. Inc. (Beijing, China). The mice were randomly separated with 10 mice in each group. The indicated cells were suspended in DMEM mixed with Matrigel at a ratio of 1:1 and then inoculated subcutaneously into the flanks of mice at 1 × 10^6^ cells/mouse. The protocol was carried out in accordance with the National Institutes of Health Guidelines for the Care and Use of Laboratory Animals (NIH Publications No. 8023, revised 1978). Mice were euthanized, the size and incidence of subcutaneous tumors were recorded.

### Chromatin immunoprecipitation (ChIP)

The ChIP assay was performed using a SimpleChIP Plus Sonication Chromatin IP Kit (Cell Signaling Technology, Danvers, MA) according to the manufacturer’s instructions with minor modifications. Immunoprecipitated DNA and input were then purified and subjected to quantitative PCR using primers specifically targeting the miR-199a-3p promoter region that encompasses the c-Jun binding site. PCR products were separated on agarose gels and visualized by ethidium bromide staining. The enrichment value was calculated relative to the input and ratio to IgG. All reactions were performed in triplicate.

### Statistical analyses

Statistical analyses were performed using SPSS software (version 21.0, IBM Corp, Armonk, NY, USA) or GraphPad Prism software (version 6.01, GraphPad Software Inc., San Diego, CA). Data were expressed as the mean ± SD or mean mean ± SEM as indicated in figure legends. Continuous variables were compared with the Student’s *t* test or the Mann–Whitney U test when applicable. Categorical variables were compared with Pearson’s χ^2^ or Fisher’s exact test. Correlations were determined by the Pearson correlation coefficient. Survival was calculated according to the Kaplan–Meier method and compared using the log-rank test. A two-sided *P* value of less than 0.05 was considered statistically significant.

Further methods used can be found in Supplementary Data.

### Significance

This study provides novel evidences linking the coexistence of hepatitis B virus X protein and transforming growth factor beta 1 with miR-199a-3p in the malignant transformation of HPCs.

## Supplementary information

Supplementary materials and methods

Supplementary figure legends

Supplementary Tables

Figure S1

Figure S2

Figure S3

Figure S4

Figure S5

Figure S6
