# Supplementary material for: TGF-β1 accelerates the hepatitis B virus X-induced malignant transformation of hepatic progenitor cells by upregulating miR-199a-3p
Source: Oncogene. 2019 Nov 18;39(8):1807–20. doi: 10.1038/s41388-019-1107-9 (PMC7033045; doi:10.1038/s41388-019-1107-9)
Supplement: Supplementary file 1 — Supplementary materials and methods [file 41388_2019_1107_MOESM1_ESM.docx]

**Supplementary materials and methods**

***Plasmids and reagents***

The control vector pLenti-CMV-GFP-Puro, pcDNA3.1, and packaging plasmids psPAX2/pMD2.G were purchased from Addgene. The HBx expression contrast vector (pLenti-HBx) was generated by inserting the HBx coding region into *Xba*I–*BamH*I sites in 3×FLAG-pLenti-CMV-GFP-Puro. pcDNA3.1-HBx was constructed by cloning HBx into pcDNA3.1. The human miR-199a-3p promoter (–2000/–1) luciferase reporter construct was purchased from GeneChem Technologies (Shanghai, China). Various lengths of miR-199a-3p promoter and c-Jun response element mutant luciferase reporter constructs were generated using the ClonExpress II One Step Cloning Kit (Vazyme, Nanjing, China) following the manufacturer’s instructions. All sequences were verified by DNA sequencing. miR-199a-3p mimics, mimic negative controls, miR-199a-3p inhibitor, inhibitor negative controls and siRNAs were ordered from RiboBio (Guangzhou, China). The miR-199a-3p overexpressing or knockdown lentivirus (LVCON320-miR-199a, LVCON464-miR-199a-3p-inhibition), and their corresponding control vectors were purchased from GeneChem Technologies (Shanghai, China). Recombinant human TGF-β1 was purchased from BioLegend (San Diego, CA). The sp600125 was purchased from Tocris (Bristol, UK).

***Immunohistochemical staining***

Tissue sections were deparaffinized in xylene, rehydrated with ethanol and subjected to antigen retrieval in boiling citrate buffer for 15 min. After peroxide block, the section was incubated with primary antibody diluted by different folds at 4℃ overnight. The section was then washed 3 times in PBST and treated with secondary antibody (Dako, Denmark) for 1 hour at room temperature. After washing, the peroxidase reaction was developed with diaminobenzidine (DAB, Dako, Denmark).

***Immunofluorescence staining***

Cells were seeded onto coverslips in a 24-well culture plate. After attachment, cells were washed three time with PBS and then fixed with 4% paraformaldehyde for 15 min. Fixed cells were permeabilized with 0.1% Triton X-100 for 15 min and blocked with 5% bovine serum albumin for 1 h. For staining, cells were incubated with primary antibodies at 4℃ overnight, followed by secondary Alexa 488 or 546-conjugated anti-mouse or rabbit antibody (Invitrogen, Carlsbad, CA) for 1 h. Finally, cover slips were incubated with DAPI (Sigma) for 5 min and immunofluorescent images were captured using an inverted fluorescent microscope.

***Spheroid formation***

For spheroid assays, single cell suspensions of 1,000 cells were seeded in 24-well ultra-low attachment Microplates (Corning, NY, USA) and cultured in serum-free DMEM/F12 medium supplemented with 20 μg/mL B27, 20 ng/mL EGF and bFGF, 5 μg/mL insulin, 0.5 μg/mL hydrocortisone, and 4 μg/mL heparin. Every 3 days, 200 μl of fresh media was added. The number of spheroids that are larger than 50 μm was counted under microscopy 2 weeks after seeding, and representative images were acquired.

***Wound healing assay***

Cell were grown to 95% confluence and monolayer were scratched a line with a 10 μl pipette tip. The images were captured at 0h, 12h and 24h after the scratch using a light microscopy. Photographs of 6 random fields across three replicate wells were captured for quantification analysis. The distance of migration was measured using Image-Pro Plus 6.0 software (Media Cybernetics Inc, Bethesda, USA).

***Cell migration and invasion assay***

Cell motility was assessed by cell migration and invasion assays using Transwell chambers (8μm pore size, Corning, NY, USA) with or without Matrigel (BD Biosciences, CA, USA). For the invasion assay, the chambers were precoated with 50 μl 1:4 mixture of Matrigel and DMEM for 2 h. Cells were plated at a density of 10^5^ per well in the upper chamber without serum, and 600 μl culture medium containing 10% FBS was added to the lower chamber. After incubation for 24-48 h, cells remaining on the upper surface of the membrane were removed with a cotton swab. The invasive cells attached to the lower surface of the membrane were fixed and stained. Photographs of 6 random fields across three replicate wells were captured for quantification analysis. Cells numbers were counted by Image-Pro Plus 6.0 software (Media Cybernetics Inc, Bethesda, USA).

***Luciferase reporter assay***

Huh7 cells were seeded at a density of 10^5^ cells per well in 24-well plates and allowed to settle for 24 h. They were then transiently transfected with pcDNA3.1-vector or pcDNA3.1-HBx. Each well was also cotransfected with the Firefly luciferase reporter contrast together with the pRL-TK Renilla luciferase construct (Promega) for the normalization of transfection efficiency. Transfections were performed using Lipofectamine 2000. After 6-8 h, the transfection medium was replaced with fresh medium with or without TGF-β1. Cells were lysed 48 h post-transfection and were assayed sequentially for Firefly and Renilla luciferase using the Dual-Luciferase Reporter Assay System (E1910, Promega) with a Glo/Max 20/20 Luminometer (Promega), according to the manufacturer’s instructions. Relative light units were calculated as the ratio of Firefly luciferase activity to Renilla luciferase activity as described previously.

***Chromatin immunoprecipitation***

Briefly, cells were crosslinked with 1% formaldehyde for 10 min at room temperature. After glycine quenching, samples were lysed in 1 ml of SDS buffer, supplemented with complete protease inhibitors. The lysates were incubated for 10 min on ice and sonicated to shear DNA. Chromatin extracts containing DNA fragments with an average of 250-1000 bp were incubated with anti-c-Jun antibody or rabbit IgG at 4℃ overnight with rotation. Immunocomplexes were captured by incubating 30 μl of Protein G Magnetic Beads for 2 h at 4℃ followed by a number of washes. Elutes were subjected to reverse cross-linking at 65℃ for 16 h, and then digested with RNase A for 2 h at 37℃ and proteinase K for 2 h at 65℃. Immunoprecipitated DNA and input were then purified and subjected to qPCR.
