# Supplementary material for: TGF-β1 accelerates the hepatitis B virus X-induced malignant transformation of hepatic progenitor cells by upregulating miR-199a-3p
Source: Oncogene. 2019 Nov 18;39(8):1807–20. doi: 10.1038/s41388-019-1107-9 (PMC7033045; doi:10.1038/s41388-019-1107-9)
Supplement: Supplementary file 2 — Supplementary figure legends [file 41388_2019_1107_MOESM2_ESM.docx]

**Supplementary Figure legends**

**Figure S1.** (A) Representative staining images are shown in tumor areas of a low or high expression patient sample. Scale bar, 100×=200 μm, 400×=50 μm. (B) Dot density plots illustrate the relative expression levels of HBx, TGF-β1, CD90 and EpCAM in 119 liver cancer samples with or without vascular invasion. *P* values were calculated by Mann-Whitney U test, **P*<0.05, ** *P*< 0.01, *** *P*<0.001.

**Figure S2.** (A) LE/6 cells were transfected with lentivirus-HBx. Stable overexpression of HBx in LE/6 cells were confirmed by western blot. (B) Stable overexpression of HBx in LE/6-HBx and LE/6-HBx+T cells were verified by qRT-PCR. (C) Immunofluorescence images of the indicated cell lines stained with OV-6 (red), CD133 (green) and EpCAM (red) are shown. Nuclei were visualized with DAPI (blue). Scale bar, 200 μm. (D) Histogram shows quantification of the number of mice with xenografted tumors upon subcutaneous injection of indicated cells (n=10). (E) HE staining and IHC staining with anti-AFP, anti-CD90 and anti-CK19 antibody of xenografted tumors are shown. Scale bar, 200×=100 μm.

**Figure S3.** (A) Wound healing assay was performed to measure the migration ability of the four cell lines as indicated. Representative images were obtained at time point 0 h, 12 h and 24 h. Scale bar, 1000 μm. (B) Transwell assay was performed to assess the cell migration and invasion abilities in the four cell lines. Representative images are shown. (C) LE/6-vec and LE/6-HBx cells were treated with TGF-β1 at 5 ng/ml or 10 ng/ml for 4 weeks. The expression of p-ERK, ERK, p-Smad3, Smad3, p-Smad2, Smad2 was detected by western blot.

**Figure S4.** (A) Top 14 highly expressed miRNAs which were chosen from MIRNA Base 16.0 microarray were quantified by qRT-PCR in LE/6-stable transfectants with or without TGF-β1 exposure. miRNA expression was normalized to U6. (B) LE/6 cells transiently transfected with pcDNA3.1-vec or pcDNA3.1-HBx were treated with/ without TGF-β1 or sp600125 for 24h. The expression of miR-215-5p, miR-374a-5p, miR-188-5p and miR-199a-3p were examined by qRT-PCR. (C) Knockdown efficiency of c-Jun by siRNA in LE/6 was verified by western blot. The siJun 1# was used for further experiments. (D) LE/6 cells were transiently transfected with siRNA control or siJun then stimulated by HBx or TGF-β1 for 24h. The expression of miR-199a-3p was detected by qRT-PCR. n=3 per group, data represent mean±SEM, *P* values were calculated by Student’s t test. *** *P*<0.001.

**Figure S5.** A schematic representation of the 482 region of miR-199a-3p promoter that contained putative c-Jun response element was shown. Mutation of c-Jun response element was designed. Underlined regions indicate the primer sequences for ChIP assay.

**Figure S6.** (A) Migration ability of the indicated cells was assessed by wound healing assay. Representative images were obtained at time point 0 h, 12 h and 24 h. (B). Transwell assay was performed to assess the cell migration and invasion abilities of the indicated cells. Representative images are shown. (C) qRT-PCR was performed to confirm the overexpression or knockdown efficacy of miR-199a-3p. n=3 per group, data represent mean±SEM, *P* values were calculated by Student’s t test. *** *P*<0.001.
