# Supplementary material for: TGF-β1 accelerates the hepatitis B virus X-induced malignant transformation of hepatic progenitor cells by upregulating miR-199a-3p
Source: Oncogene. 2019 Nov 18;39(8):1807–20. doi: 10.1038/s41388-019-1107-9 (PMC7033045; doi:10.1038/s41388-019-1107-9)
Supplement: Supplementary file 3 — Supplementary Tables [file 41388_2019_1107_MOESM3_ESM.docx]

**Supplementary Tables**

| **Table S1. The correlation between TGF-β1, HBx, CD90, EpCAM expression and clinicopathological features in 119 primary liver cancer patients** | | | | | | | | | | | | | |
| --- | --- | --- | --- | --- | --- | --- | --- | --- | --- | --- | --- | --- | --- |
| Clinical variables | No. | TGF-β1 | | *P* | HBx | | *P* | CD90 | | *P* | EpCAM | | *P* |
|  |  | Low | High |  | Low | High |  | Low | High |  | Low | High |  |
| Age(years) |  |  |  | 0.615 |  |  | 0.157 |  |  | 0.681 |  |  | 0.157 |
| <60 | 95 | 46 | 39 |  | 51 | 44 |  | 47 | 48 |  | 51 | 44 |  |
| ≥60 | 24 | 13 | 11 |  | 9 | 15 |  | 13 | 11 |  | 9 | 15 |  |
| Gender |  |  |  | 0.636 |  |  | 0.969 |  |  | 0.325 |  |  | 0.636 |
| Male | 101 | 51 | 50 |  | 51 | 50 |  | 49 | 52 |  | 50 | 51 |  |
| Female | 18 | 8 | 10 |  | 9 | 9 |  | 11 | 7 |  | 10 | 8 |  |
| AFP(ng/ml) |  |  |  | 0.903 |  |  | 0.615 |  |  | 0.205 |  |  | 0.042 |
| <20 | 43 | 21 | 22 |  | 23 | 20 |  | 25 | 18 |  | 27 | 16 |  |
| ≥20 | 76 | 38 | 38 |  | 37 | 39 |  | 35 | 41 |  | 33 | 43 |  |
| Child-Pugh Class |  |  |  | 0.547 |  |  | 0.094 |  |  | 0.973 |  |  | 0.973 |
| A | 105 | 51 | 54 |  | 50 | 55 |  | 53 | 52 |  | 53 | 52 |  |
| B | 14 | 8 | 6 |  | 10 | 4 |  | 7 | 7 |  | 7 | 7 |  |
| Liver cirrhosis |  |  |  | 0.040 |  |  | 0.002 |  |  | 0.020 |  |  | 0.461 |
| No | 36 | 23 | 13 |  | 26 | 10 |  | 24 | 12 |  | 20 | 16 |  |
| Yes | 83 | 36 | 47 |  | 34 | 49 |  | 36 | 47 |  | 40 | 43 |  |
| Tumor size(cm) |  |  |  | 0.512 |  |  | 0.891 |  |  | 0.795 |  |  | 0.185 |
| ≤5 | 37 | 20 | 17 |  | 19 | 18 |  | 18 | 19 |  | 22 | 15 |  |
| >5 | 82 | 39 | 43 |  | 41 | 41 |  | 42 | 40 |  | 38 | 44 |  |
| Tumor number |  |  |  | 0.703 |  |  | 0.093 |  |  | 0.360 |  |  | 0.360 |
| Single | 91 | 46 | 45 |  | 42 | 49 |  | 48 | 43 |  | 48 | 43 |  |
| Multiple | 28 | 13 | 15 |  | 18 | 10 |  | 12 | 16 |  | 12 | 16 |  |
| Vascular invasion |  |  |  | 0.005 |  |  | 0.866 |  |  | 0.253 |  |  | 0.043 |
| Yes | 27 | 7 | 20 |  | 14 | 13 |  | 11 | 16 |  | 9 | 18 |  |
| No | 92 | 52 | 40 |  | 46 | 46 |  | 49 | 43 |  | 51 | 41 |  |
| Tumor differentiation | |  |  | 0.502 |  |  | 0.94 |  |  | 0.411 |  |  | 0.020 |
| Well/Moderate | 71 | 37 | 34 |  | 36 | 35 |  | 38 | 33 |  | 42 | 29 |  |
| Poor | 48 | 22 | 26 |  | 24 | 24 |  | 22 | 26 |  | 18 | 30 |  |
| TNM stage |  |  |  | 0.074 |  |  | 0.882 |  |  | 0.502 |  |  | 0.002 |
| I-II | 86 | 47 | 39 |  | 43 | 43 |  | 45 | 41 |  | 51 | 35 |  |
| III-IV | 33 | 12 | 21 |  | 17 | 16 |  | 15 | 18 |  | 9 | 24 |  |

| **Table S2. Clinical characteristics of primary liver cancer from tissue microarray samples.** | | | | | | | | |
| --- | --- | --- | --- | --- | --- | --- | --- | --- |
| No. | Sex | Age | Organ | Pathology diagnosis | Child-pugh | tumor size(cm) | tumor number | TNM Stage |
| 1 | M | 55 | Liver | hepatocellular carcinoma | A | 7.2 | 1 | I |
| 2 | M | 46 | Liver | hepatocellular carcinoma | A | 7.9 | 1 | I |
| 3 | M | 34 | Liver | hepatocellular carcinoma | A | 7 | 1 | III |
| 4 | M | 69 | Liver | hepatocellular carcinoma | A | 14.5 | 1 | I |
| 5 | M | 43 | Liver | hepatocellular carcinoma | A | 9.2 | 2 | III |
| 6 | M | 42 | Liver | hepatocellular carcinoma | A | 2.5 | 1 | I |
| 7 | M | 66 | Liver | hepatocellular carcinoma | A | 2.8 | 1 | I |
| 8 | F | 42 | Liver | hepatocellular carcinoma | B | 5.4 | 1 | I |
| 9 | M | 65 | Liver | hepatocellular carcinoma | A | 8.5 | 1 | I |
| 10 | F | 51 | Liver | hepatocellular carcinoma | A | 5.3 | 1 | I |
| 11 | F | 56 | Liver | hepatocellular carcinoma | A | 6 | 1 | I |
| 12 | M | 65 | Liver | intrahepatic cholangiocarcinoma | A | 4.2 | 1 | I |
| 13 | M | 42 | Liver | hepatocellular carcinoma | A | 7.9 | 4 | III |
| 14 | M | 57 | Liver | hepatocellular carcinoma | A | 9.4 | 1 | I |
| 15 | M | 50 | Liver | hepatocellular carcinoma | A | 13.6 | 1 | I |
| 16 | F | 59 | Liver | hepatocellular carcinoma | A | 4 | 1 | I |
| 17 | M | 33 | Liver | hepatocellular carcinoma | A | 7 | 2 | III |
| 18 | F | 61 | Liver | hepatocellular carcinoma | A | 10 | 1 | I |
| 19 | M | 44 | Liver | hepatocellular carcinoma | A | 4.1 | 1 | I |
| 20 | M | 55 | Liver | hepatocellular carcinoma | A | 4 | 1 | I |
| 21 | M | 57 | Liver | hepatocellular carcinoma | B | 10 | 4 | IV |
| 22 | M | 56 | Liver | hepatocellular carcinoma | B | 6.8 | 1 | III |
| 23 | M | 45 | Liver | hepatocellular carcinoma | A | 6.9 | 2 | III |
| 24 | M | 49 | Liver | hepatocellular carcinoma | A | 8.1 | 1 | I |
| 25 | M | 40 | Liver | hepatocellular carcinoma | A | 10 | 4 | III |
| 26 | M | 54 | Liver | intrahepatic cholangiocarcinoma | A | 7.3 | 3 | IV |
| 27 | M | 53 | Liver | intrahepatic cholangiocarcinoma | A | 4.5 | 4 | II |
| 28 | M | 36 | Liver | hepatocellular carcinoma | A | 16 | 3 | III |
| 29 | M | 49 | Liver | hepatocellular carcinoma | A | 2.4 | 1 | I |
| 30 | M | 50 | Liver | hepatocellular carcinoma | A | 7.6 | 1 | I |
| 31 | M | 46 | Liver | hepatocellular carcinoma | B | 7.1 | 1 | I |
| 32 | M | 38 | Liver | hepatocellular carcinoma | A | 4.7 | 1 | I |
| 33 | M | 56 | Liver | hepatocellular carcinoma | A | 9.3 | 4 | III |
| 34 | M | 47 | Liver | hepatocellular carcinoma | A | 6 | 1 | I |
| 35 | M | 58 | Liver | hepatocellular carcinoma | B | 8.4 | 1 | I |
| 36 | M | 47 | Liver | hepatocellular carcinoma | A | 4.8 | 1 | I |
| 37 | M | 61 | Liver | hepatocellular carcinoma | A | 4 | 1 | I |
| 38 | M | 50 | Liver | intrahepatic cholangiocarcinoma | A | 20.7 | 1 | I |
| 39 | M | 42 | Liver | hepatocellular carcinoma | A | 14.4 | 4 | III |
| 40 | F | 57 | Liver | hepatocellular carcinoma | A | 3.4 | 1 | I |
| 41 | M | 42 | Liver | hepatocellular carcinoma | A | 14 | 1 | I |
| 42 | M | 34 | Liver | hepatocellular carcinoma | A | 7.9 | 2 | III |
| 43 | M | 28 | Liver | hepatocellular carcinoma | A | 3.2 | 2 | II |
| 44 | M | 57 | Liver | hepatocellular carcinoma | A | 3.2 | 1 | I |
| 45 | M | 57 | Liver | hepatocellular carcinoma | A | 5.2 | 2 | III |
| 46 | M | 55 | Liver | hepatocellular carcinoma | A | 10 | 1 | I |
| 47 | F | 36 | Liver | hepatocellular carcinoma | B | 14.1 | 4 | III |
| 48 | M | 51 | Liver | hepatocellular carcinoma | A | 3.5 | 1 | I |
| 49 | M | 66 | Liver | hepatocellular carcinoma | A | 4.6 | 1 | I |
| 50 | M | 45 | Liver | hepatocellular carcinoma | A | 3.9 | 1 | I |
| 51 | M | 37 | Liver | hepatocellular carcinoma | A | 11.9 | 1 | I |
| 52 | M | 39 | Liver | hepatocellular carcinoma | A | 3.9 | 1 | III |
| 53 | F | 46 | Liver | hepatocellular carcinoma | A | 15 | 1 | I |
| 54 | M | 62 | Liver | hepatocellular carcinoma | A | 11.1 | 1 | I |
| 55 | M | 76 | Liver | hepatocellular carcinoma | A | 5.8 | 1 | I |
| 56 | F | 63 | Liver | intrahepatic cholangiocarcinoma | A | 12 | 1 | I |
| 57 | F | 62 | Liver | hepatocellular carcinoma | A | 3.7 | 1 | I |
| 58 | M | 66 | Liver | hepatocellular carcinoma | A | 8.5 | 1 | I |
| 59 | M | 58 | Liver | hepatocellular carcinoma | A | 3.3 | 1 | I |
| 60 | M | 41 | Liver | hepatocellular carcinoma | A | 3.3 | 1 | I |
| 61 | M | 45 | Liver | hepatocellular carcinoma | A | 9.6 | 1 | I |
| 62 | M | 48 | Liver | hepatocellular carcinoma | A | 9.2 | 1 | I |
| 63 | M | 58 | Liver | hepatocellular carcinoma | B | 3.1 | 1 | I |
| 64 | M | 46 | Liver | hepatocellular carcinoma | A | 9.9 | 1 | I |
| 65 | M | 49 | Liver | hepatocellular carcinoma | A | 3.9 | 1 | I |
| 66 | M | 64 | Liver | hepatocellular carcinoma | A | 5 | 1 | I |
| 67 | F | 28 | Liver | hepatocellular carcinoma | A | 8.7 | 4 | III |
| 68 | M | 47 | Liver | hepatocellular carcinoma | A | 5.6 | 1 | I |
| 69 | M | 32 | Liver | hepatocellular carcinoma | A | 2.9 | 1 | I |
| 70 | M | 43 | Liver | hepatocellular carcinoma | A | 6.9 | 1 | I |
| 71 | M | 56 | Liver | hepatocellular carcinoma | A | 11.6 | 2 | III |
| 72 | M | 49 | Liver | hepatocellular carcinoma | A | 16.6 | 4 | III |
| 73 | M | 41 | Liver | hepatocellular carcinoma | A | 7.3 | 1 | IV |
| 74 | M | 40 | Liver | hepatocellular carcinoma | A | 11.4 | 1 | I |
| 75 | M | 58 | Liver | hepatocellular carcinoma | B | 9.7 | 1 | III |
| 76 | M | 23 | Liver | hepatocellular carcinoma | A | 13.9 | 1 | I |
| 77 | M | 60 | Liver | hepatocellular carcinoma | A | 3.2 | 1 | I |
| 78 | M | 47 | Liver | hepatocellular carcinoma | A | 11.3 | 1 | I |
| 79 | M | 46 | Liver | hepatocellular carcinoma | B | 7.6 | 6 | III |
| 80 | M | 67 | Liver | hepatocellular carcinoma | A | 11.2 | 1 | I |
| 81 | M | 66 | Liver | hepatocellular carcinoma | A | 7.2 | 1 | I |
| 82 | M | 56 | Liver | hepatocellular carcinoma | B | 6.1 | 1 | III |
| 83 | M | 49 | Liver | hepatocellular carcinoma | A | 10.7 | 1 | I |
| 84 | M | 69 | Liver | hepatocellular carcinoma | A | 7.3 | 1 | I |
| 85 | M | 57 | Liver | hepatocellular carcinoma | A | 5.1 | 1 | I |
| 86 | M | 47 | Liver | hepatocellular carcinoma | A | 8.5 | 1 | I |
| 87 | M | 47 | Liver | hepatocellular carcinoma | A | 9.2 | 1 | III |
| 88 | M | 36 | Liver | hepatocellular carcinoma | B | 11 | 4 | III |
| 89 | M | 40 | Liver | hepatocellular carcinoma | A | 8.2 | 1 | III |
| 90 | M | 51 | Liver | hepatocellular carcinoma | B | 2.5 | 1 | I |
| 91 | F | 56 | Liver | hepatocellular carcinoma | A | 4.3 | 1 | I |
| 92 | M | 34 | Liver | hepatocellular carcinoma | A | 11.5 | 1 | I |
| 93 | M | 44 | Liver | hepatocellular carcinoma | B | 5.7 | 1 | I |
| 94 | M | 43 | Liver | hepatocellular carcinoma | A | 7 | 1 | I |
| 95 | M | 59 | Liver | hepatocellular carcinoma | A | 7 | 1 | I |
| 96 | M | 47 | Liver | hepatocellular carcinoma | A | 9.2 | 4 | III |
| 97 | M | 32 | Liver | hepatocellular carcinoma | A | 3.2 | 2 | III |
| 98 | M | 41 | Liver | hepatocellular carcinoma | A | 5.2 | 2 | III |
| 99 | M | 75 | Liver | hepatocellular carcinoma | A | 4.3 | 1 | I |
| 100 | F | 36 | Liver | hepatocellular carcinoma | A | 10 | 4 | III |
| 101 | M | 61 | Liver | hepatocellular carcinoma | A | 12.5 | 1 | I |
| 102 | M | 54 | Liver | hepatocellular carcinoma | A | 4.4 | 1 | I |
| 103 | M | 51 | Liver | intrahepatic cholangiocarcinoma | A | 5 | 1 | IV |
| 104 | F | 57 | Liver | intrahepatic cholangiocarcinoma | A | 2.7 | 1 | I |
| 105 | M | 47 | Liver | hepatocellular carcinoma | A | 7.5 | 4 | III |
| 106 | M | 69 | Liver | hepatocellular carcinoma | A | 5.4 | 1 | I |
| 107 | F | 68 | Liver | hepatocellular carcinoma | A | 12 | 1 | III |
| 108 | M | 47 | Liver | hepatocellular carcinoma | A | 7 | 1 | III |
| 109 | M | 55 | Liver | hepatocellular carcinoma | A | 7 | 1 | I |
| 110 | M | 69 | Liver | hepatocellular carcinoma | A | 7.3 | 4 | III |
| 111 | F | 60 | Liver | hepatocellular carcinoma | A | 6.6 | 1 | I |
| 112 | M | 42 | Liver | hepatocellular carcinoma | A | 4.1 | 1 | I |
| 113 | M | 41 | Liver | hepatocellular carcinoma | A | 12 | 1 | I |
| 114 | M | 47 | Liver | hepatocellular carcinoma | A | 3.3 | 2 | III |
| 115 | M | 53 | Liver | hepatocellular carcinoma | A | 3 | 1 | III |
| 116 | M | 61 | Liver | hepatocellular carcinoma | A | 13.6 | 1 | III |
| 117 | M | 46 | Liver | hepatocellular carcinoma | A | 5 | 1 | III |
| 118 | F | 50 | Liver | hepatocellular carcinoma | A | 9.1 | 1 | I |
| 119 | F | 51 | Liver | hepatocellular carcinoma | A | 4.2 | 1 | I |

| **Table S3. List of antibodies used in this study** | | | | | | |
| --- | --- | --- | --- | --- | --- | --- |
| Antibody | WB | IHC | IF | Specificity | Company | Catalog Number |
| HBx | 1:500 | 1:200 | / | Mouse monoclonal | Santa Cruz Biotechnology | sc-57760 |
| TGF-β1 | / | 1:200 | / | Rabbit polyclonal | Proteintech | 21898-1-AP |
| CD90 | 1:1000 | 1:200 | / | Rabbit monoclonal | Epitomics | 2694-1 |
| EpCAM | 1:1000 | 1:200 | 1:100 | Mouse monoclonal | Cell Signaling Technology | #2929 |
| CD133 | 1:1000 | / | 1:50 | Rabbit polyclonal | Proteintech | 18470-1-AP |
| CK19 | 1:1000 | 1:500 | / | Rabbit polyclonal | Proteintech | 10712-1-AP |
| AFP | 1:2000 | 1:200 | / | Rabbit polyclonal | Proteintech | 14550-1-AP |
| β-actin | 1:1000 | / | / | Rabbit polyclonal | Cell Signaling Technology | #4967 |
| OV-6 | / | / | 1:50 | Mouse monoclonal | R&D systems | MAB2020 |
| ZO-1 | 1:1000 | / | / | Rabbit polyclonal | Proteintech | 21773-1-AP |
| N-cadherin | 1:1000 | / | / | Mouse monoclonal | BD Biosciences | 610921 |
| E-cadherin | 1:2000 | / | / | Mouse monoclonal | BD Biosciences | 610182 |
| Fibronectin | 1:5000 | / | / | Mouse monoclonal | BD Biosciences | 610077 |
| ZEB1 | 1:1000 | / | / | Rabbit polyclonal | Proteintech | 21544-1-AP |
| Snail | 1:1000 | / | / | Rabbit polyclonal | Proteintech | 13099-1-AP |
| p-JNK | 1:1000 | / | / | Rabbit polyclonal | Millipore | 07-175 |
| JNK | 1:1000 | / | / | Rabbit monoclonal | Cell Signaling Technology | #9258 |
| p-P38 | 1:1000 | / | / | Rabbit monoclonal | Cell Signaling Technology | #9215 |
| P38 | 1:1000 | / | / | Rabbit monoclonal | Cell Signaling Technology | #8690 |
| p-ERK | 1:1000 | / | / | Rabbit monoclonal | Cell Signaling Technology | #4370 |
| ERK | 1:1000 | / | / | Mouse monoclonal | Santa Cruz Biotechnology | sc-514302 |
| p-c-Jun (Ser73) | 1:1000 | / | / | Rabbit monoclonal | Cell Signaling Technology | #3270 |
| c-Jun | 1:1000 | / | / | Rabbit monoclonal | Cell Signaling Technology | #9165 |
| p-Smad2 | 1:500 | / | / | Rabbit polyclonal | Millipore | AB3849-I |
| p-Smad3 | 1:500 | / | / | Rabbit polyclonal | Millipore | 07-1389 |
| Smad2 | 1:1000 | / | / | Rabbit monoclonal | Cell Signaling Technology | #5339 |
| Smad3 | 1:1000 | / | / | Rabbit monoclonal | Cell Signaling Technology | #9523 |

| **Table S4. List of primers used in this study** | | |
| --- | --- | --- |
| Primer | Forward Primer (5′-3′) | Reverse Primer (5′-3′) |
| **Primers for luciferase assay** | | |
| pGL4.17-2000 | CTGGCCTAACTGGCCGGTACCCAACACAACTGCCCCTCGC | CCAGATCTTGATATCCTCGAGGGGGCCGGCTATCCATCC |
| pGL4.17-1175 | CTGGCCTAACTGGCCGGTACCCAACACAACTGCCCCTCGC | CCAGATCTTGATATCCTCGAGAGCCTGGCAGGCTAATCCTG |
| pGL4.17-825 | CTGGCCTAACTGGCCGGTACCCTAGGCAAAGAAGTAGGGCAGAGC | CCAGATCTTGATATCCTCGAGGGGGCCGGCTATCCATCC |
| pGL4.17-833 | CTGGCCTAACTGGCCGGTACCCAACACAACTGCCCCTCGC | CCAGATCTTGATATCCTCGAGCTGTGGCTGCTCCTGAACTTG |
| pGL4.17-482 | CTGGCCTAACTGGCCGGTACCAAGCCCAGTCCTCCCCACA | CCAGATCTTGATATCCTCGAGAGCCTGGCAGGCTAATCCTG |
| pGL4.17-482-mutant | CACAGACACACAGGAGAAGGCAGCGGGGGCTC | TCTCCTGTGTGTCTGTGGCTGCTCCTGAACTTG |
| **Primers for CDS cloning** | | |
| pcDNA3.1-Flag-HBx | CTTGGTACCGAGCTCGGATCCATGGCTGCTAGGCTGTGCTG | TGCTGGATATCTGCAGAATTCTTAGGCAGAGGTGAAAAAGTTGC |
| pLenti-Flag-HBx | CATAGAAGACACCGACTCTAGACGCCACCATGGCTGCTAGGCTGTGCTG | CTTTGTAGTCAGCCCGGGATCC GGCAGAGGTGAAAAAGTTGCA |
| **Primers for RT-PCR and qRT-PCR** | | |
| HBx | TGCTGCCAACTGGATCCTG | ATGCCTCAAGGTCGGTCGT |
| GAPDH | ATCACCATCTTCCAGGAGCGA | CCTTCTCCATGGTGGTGAAGAC |
| hsa-miR-215-5p | GCGCGATGACCTATGAATTGACAGAC | universal primers |
| hsa-miR-374a-5p | GCGCGCGTTATAATACAACCTGATAAGTG |  |
| hsa-miR-23b-3p | GGATCACATTGCCAGGGATTAC |  |
| hsa-miR-18a-5p | GCGTAAGGTGCATCTAGTGCAGATAG |  |
| hsa-miR-4271 | GGGGGAAGAAAAGGTGGGG |  |
| hsa-miR-1471 | ATTTACGTGTGGAGCCAGGTGT |  |
| has-miR-320a-3p | AACGGAAAAGCTGGGTTGAGAG |  |
| hsa-miR-188-5p | CGGTTTACTTGCATGGTGGAGG |  |
| hsa-miR-1183 | CACTGTAGGTGATGGTGAGAGTGG |  |
| hsa-miR-3610 | GAATCGGAAAGGAGGCGCC |  |
| hsa-miR-199a-3p | CGCACAGTAGTCTGCACATTGGTTA |  |
| hsa-miR-454-3p | CCGCGTAGTGCAATATTGCTTATAGGGT |  |
| has-miR-423-5p | ATTTACGGCAGAGAGCGAGAC |  |
| hsa-miR-151-5p | CGTCGAGGAGCTCACAGTCTAGT |  |
| U6 | CTCGCTTCGGCAGCACA |  |
| **Primers for ChIP assay** | | |
| miR-199a-3p promoter c-Jun binding site | CAGCACAAAGCCTAACCT | CATCACCTCAACCATGCT |
