# Supplementary figures and images for: TGF-β1 accelerates the hepatitis B virus X-induced malignant transformation of hepatic progenitor cells by upregulating miR-199a-3p
Source: Oncogene. 2019 Nov 18;39(8):1807–20. doi: 10.1038/s41388-019-1107-9 (PMC7033045; doi:10.1038/s41388-019-1107-9)

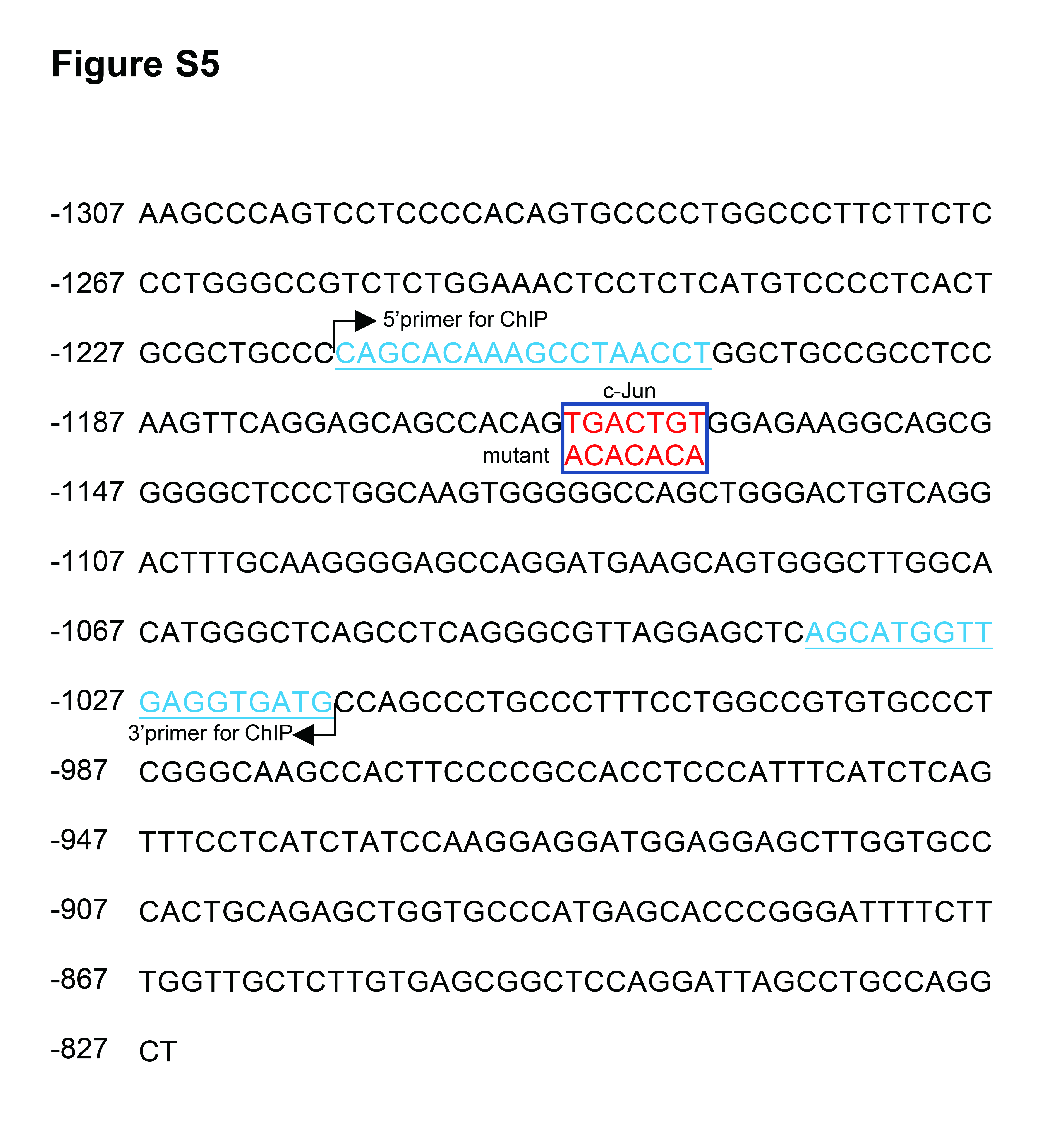

Supplement: Supplementary file 8 — Figure S5 [file 41388_2019_1107_MOESM8_ESM.tif]
